# Penetrating Orbital Injury; a Case Report and Treatment Algorithm

**Published:** 2020-03-19

**Authors:** Mehrdad Dehghanpour Barouj, Reza Tabrizi, Parsa Behnia, Mohammad Amir Alizadeh Tabrizi, Mahtab Kheirkhahi

**Affiliations:** 1 Oral and Maxillofacial Surgery Department, Shahid Beheshti University of Medical Sciences, School of Dentistry, Tehran, Iran.; 2 oral and maxillofacial radiologist, Shahid Beheshti University of Medical Sciences, School of Dentistry, Tehran, Iran.

**Keywords:** Wounds and injuries, orbit, head injuries, penetrating, optic nerve injuries

## Abstract

Penetrating orbital trauma (POT) consists of high and low velocity penetrating injuries that may lead to severe consequences such as visual impairment and globe tearing. It has been reported to make up 30% to 50% of all orbital injuries. POT requires a multidisciplinary approach due to complex orbital injury, which involves eye function, brain injury, and facial aesthetics. In this report, we presented a case of POT due to knife injury in which the knife blade was removed and bleeding was controlled, the patient’s general condition after surgery was good, but the vision of the right eye was lost.

## Introduction

 Penetrating orbital trauma (POT) consists of high and low velocity penetrating injuries that may lead to severe consequences such as visual impairment and globe tearing. Penetrating orbital trauma has been reported to make up 30% to 50% of all orbital injuries (1). POT requires a multidisciplinary approach due to complex orbital injury, which involves eye function, brain injury, and facial aesthetics (2). Clinical decision-making in management of patients with POT is challenging. The prognosis of treatments depends on the severity of the injury and the involvement of orbital structures (3). The foreign body impacted in orbit may be organic or inert. Organic foreign bodies like wood need to be removed at the earliest due to the high risk of infection associated. Inert materials like glass, plastic or steel are associated with lower risk of infection, and a decision to remove them should be based on factors like site of impingement, size of the foreign body, potential of secondary injuries and hemostasis (4). The physical characteristics of the foreign body like mass and shape are also of prognostic importance (5). In this report, we presented a case of POT due to knife injury in which the knife blade was removed and bleeding was controlled, the patient’s general condition after surgery was good, but the vision of the right eye was lost.

## Case presentation

**Figure 1:**
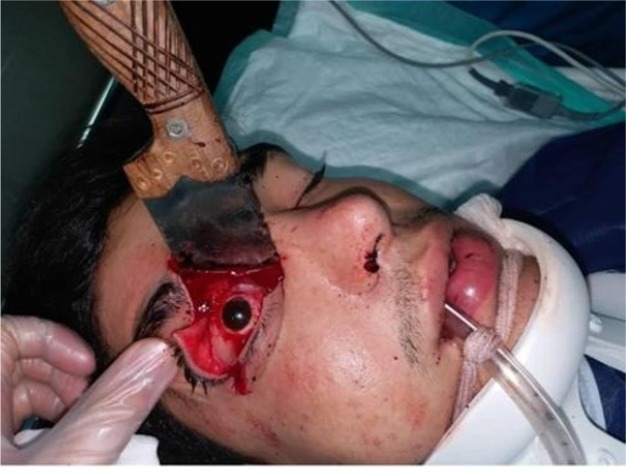
A knife penetrating the right orbit

An 18-year-old man (weight = 70 kg) presented to the emergency department of Sevom-e-Shaban Hospital, Tehran, Iran, after being stabbed with a huge knife penetrating to his right orbit with irregular laceration and wound on his right upper eyelid (Figure1). The incidence took place on the same day at midnight. After providing primary care, spiral facial and orbital computed tomography scans were obtained and the patient was transferred to the department of Oral and Maxillofacial Surgery, Imam Hossein Hospital, Tehran, Iran. He was admitted with the Glasgow Coma Scale score of 15, dizziness, intact airway, pulse rate of 78 per min, blood pressure 132/75 mmHg. 

The patient was intubated in the cardiopulmonary resuscitation (CPR) room because he was too agitated. His past medical history showed previous hospitalization and general anesthesia due to rhinoplasty in 2016. He was under daily medication with Pregabalin 50 mg capsule without any history of allergy and any other health problem. His habitual history revealed a history of smoking (1.5 pack-year), alcohol, and drug abuse (cannabis).

Clinical examination showed intact scalp, 1cm laceration in the forehead, and superior and inferior orbital rim as well as the medial canthus of right eye were ruptured. The ophthalmologic examination demonstrated eyelid laceration, abnormal pupil reflex, abnormal extraocular muscle function, no periorbital ecchymosis, no exophthalmos, no enophthalmos, the vision was lost, and there was subconjunctival hemorrhage in the right eye.

**Figure 2:**
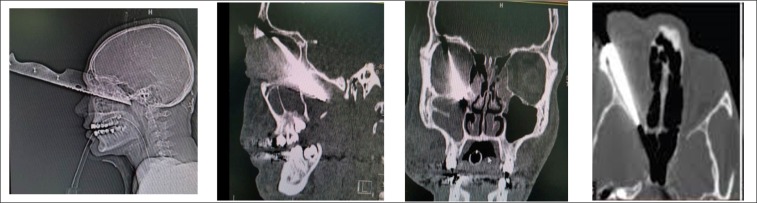
CT scan view shows orbital and skull base involvement

Spiral facial computed tomography (CT) Scan and three-dimensional images showed a foreign body (the knife blade) entering the right orbital region, which had not penetrated to the skull. Superior and inferior orbital rim, maxilla, zygomatic arch, and mandible were intact (Figure 2). Orbital CT scans were useful for predicting visual prognosis.

After comprehensive evaluation of systemic condition and stabilization of the patient, a multidisciplinary approach with oral and maxillofacial surgery and ophthalmology teams was applied. The globe was retracted with a malleable retractor. Then some of the bones around the knife blade were removed with long shank round bur, and the knife was removed very carefully under general anesthesia. Bleeding was successfully controlled. There was not any globe rupture, but the optic nerve was cut; therefore, the right eye was not exenterated. The medial tendon lacerations were repaired using monocryl 6-0 sutures, and dacryocystorhinostomy was done by ophthalmologic team without any complication. Then skin was sutured with 6-0 monofilament nylon. The patient remained in the intensive care unit for 24 hours and was then extubated. The patient was maintained on intravenous clindamycin (600 mg every eight hours) systemically with Erythromycin ointment twice daily, and betamethasone IV (1% every 12h).

**Figure 3:**
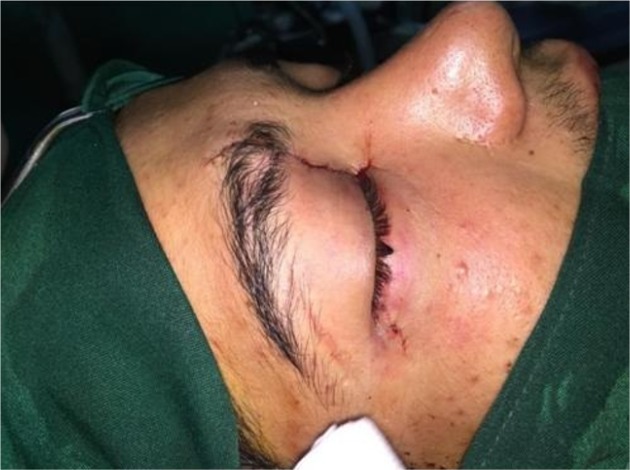
Patient after removing the knife

Figure 3 shows the patient after trauma management. In clinical examination, cranial nerves other than optic nerve (V, VII, VIII, XII) were intact. The vision of right eye was lost due to transection of the right optic nerve. 

## Discussion

POTs caused by sharp instruments or foreign bodies are all potentially serious and should be managed as an emergency. POT causes immediate damage to the eye, post-traumatic iridocyclitis, and traumatic cataract, and might lead to infection and, sympathetic ophthalmitis (5). Missile and non-missile injuries could result in POT. Metallic (magnetic, non-magnetic) and non-metallic (plants, plastics, glass, etc.) orbital foreign bodies are associated with POT (6). Penetrating or perforating injuries could be caused by foreign bodies (5). Generally, foreign bodies that are placed intraocularly are penetrating and can cross through the cornea (65%), sclera (25%), or the limbus (10%) (7). These foreign bodies are usually seen in the posterior segment (58-88%) and less frequently in the anterior chamber (10-15%) or the lens (2-8%) (8, 9).

**Figure 4:**
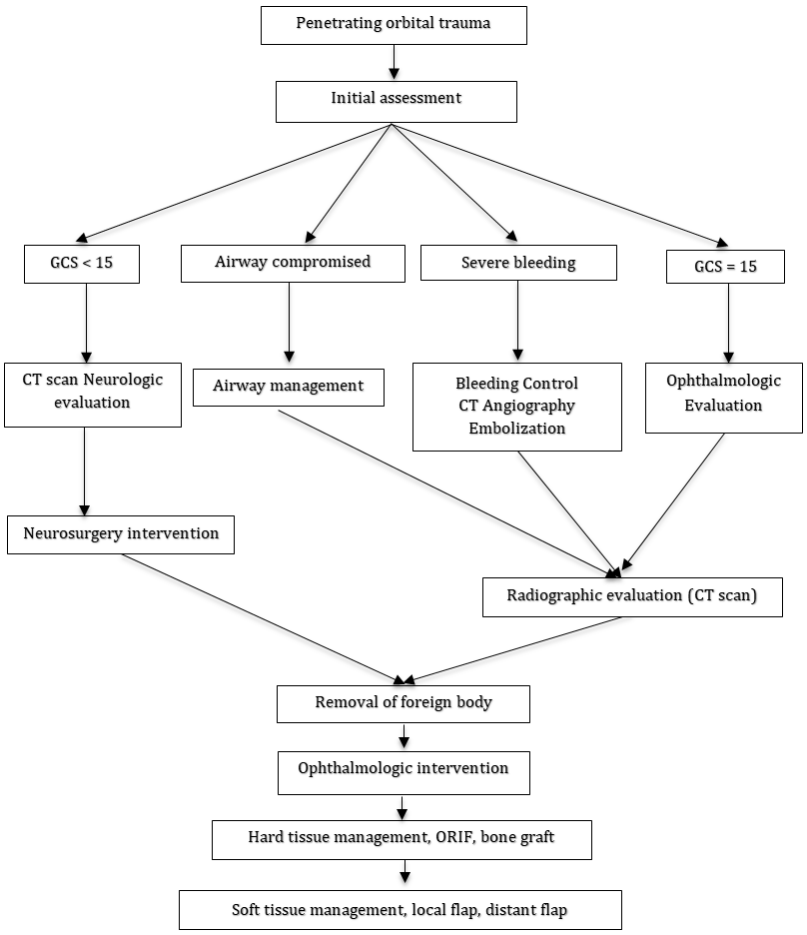
The algorithm of penetrating orbital trauma treatment. GCS: Glasgow coma scale; CT: computed tomography; ORIF: open reduction internal fixation

The presence of foreign bodies increases the risk of post-traumatic endophthalmitis (5). Management of POT depends on involvement of vital structures such as the globe and brain. A complete physical examination, including full neurological and/or ophthalmological examinations, is essential for diagnosis and treatment of a patient diagnosed with POT. All patients should undergo a comprehensive exam to rule out any penetrating intracranial injury (10, 11). CT scan imaging should be considered as the primary imaging method of choice in the emergency department. Plain radiographic imaging is recommended when a CT scan is not available. While CT can easily detect metallic foreign bodies, its value in detection of wooden foreign bodies is questionable. For the evaluation of wooden objects, contrast-enhanced magnetic resonance imaging (MRI) is suggested (12). MRI is also useful for differentiating the object from the surrounding air and fatty tissue, particularly in orbital injuries. CT angiography or magnetic resonance (MR) angiography is indicated when there is evidence of bleeding or a possible vascular injury, due to either the location and path of the foreign body or sign of a hematoma on CT scan (13).

The principles of advanced trauma support must be followed like any trauma case. Assessment of POT should be done after stabilization. Removal of the foreign body is postponed until physical examination and full radiological evaluation are completed. Early removal of the foreign body outside of the controlled situation and the operating room may lead to a fatal hemorrhage (12, 14). Intracranial pressure should be monitored when accurate neurologic assessment cannot be done. When the scalp is not involved, in those with no significant intracranial injury, POT may be treated by simple wound care and closure of the entrance wounds (11). Broad-spectrum antibiotics should be prescribed to prevent central nervous system (CNS) infection. Clinicians should minimize the degree of debridement and try to provide watertight dural and scalp closure in order to decrease Cerebrospinal fluid (CSF) leakage and infections. In the absence of intracranial or ocular injuries, simply removal of foreign body and soft tissue closure is indicated. When POT is associated with soft tissue loss, like in missile injuries, soft tissue reconstruction with local and distant flaps is recommended. Figure 4 shows the authors’ recommended algorithm for POT management.

## Conclusion

In summary, POT prognosis depends on the severity of injury and intracranial and ocular involvement. Proper diagnosis and appropriate treatment also affect the prognosis of treatment. 
